# Optimization of Window Study Endpoints in Endometrial Cancer

**DOI:** 10.3389/fonc.2019.00428

**Published:** 2019-05-29

**Authors:** Sarah J. Kitson, Zoe Maskell, Vanitha N. Sivalingam, Joseph Shaw, Emma J. Crosbie

**Affiliations:** ^1^Division of Cancer Sciences, Faculty of Biology, Medicine and Health, University of Manchester, St. Mary's Hospital, Manchester, United Kingdom; ^2^Department of Histopathology, Manchester Academic Health Science Centre, Manchester University NHS Foundation Trust, Manchester, United Kingdom; ^3^Department of Obstetrics and Gynaecology, Manchester Academic Health Science Centre, Manchester University NHS Foundation Trust, Manchester, United Kingdom

**Keywords:** endometrial cancer, window study, optimization, biomarker, expression

## Abstract

Pre-surgical window studies rely on the accurate quantification of biomarkers as surrogates of disease response. In endometrial cancer, this has traditionally involved comparing immunohistochemical expression in diagnostic endometrial biopsies with the post-treatment hysterectomy specimen. This strategy is at risk of generating erroneous results if significant hypoxia occurs during surgery or delays in fixation of tissues lead to protein loss. Immunohistochemical expression of commonly studied biomarkers in window studies were compared in pre-operative endometrial biopsies and hysterectomy specimens taken on the same day from 75 women with endometrial cancer enrolled in a clinical trial. Differences in expression were correlated with clinico-pathological variables and tissue handling. Expression of Ki-67, markers of the PI3K-Akt-mTOR, and insulin signaling pathways and hormone receptors was significantly lower in the hysterectomy specimen than the corresponding endometrial biopsy (all *p* < 0.0001). In contrast, expression of the cancer stem cell markers, CD133 and ALDH, were similar in the two specimens. The extent to which protein expression was lost in the hysterectomy specimen was closely correlated with baseline expression in the endometrial biopsy (all *p* ≤ 0.001). Bisection of the uterus prior to placement in formalin partially preserved protein expression suggesting prompt fixation is critical. These results call into question findings from earlier endometrial cancer window studies which have relied on the hysterectomy specimen for analysis and suggest a post-intervention endometrial biopsy should be included in trials going forward.

## Introduction

There is increasing interest in the use of the pre-surgical window study design to determine the efficacy of novel and repurposed cancer therapeutics in a time- and cost-effective manner. Such a strategy relies on the accurate quantification of biomarkers, which act as surrogates for longer term clinical outcomes and disease response. In endometrial cancer, this has traditionally involved comparing a baseline endometrial biopsy with tumor sampled from the hysterectomy specimen following intervention. This does, however, potentially raise a methodological issue. The endometrial biopsy samples the cancer *in situ* and is thus an accurate representation of tumor biology. The hysterectomy specimen, by contrast, is subject to a variable period of hypoxia once the uterine arteries are clamped during surgery and before it is removed from the body, followed by cold ischemia until formalin fixation occurs. Many of the biomarkers interrogated as outcome measures in endometrial cancer window studies are activated and deactivated through phosphorylation and dephosphorylation, events which have been shown to be transient and highly sensitive to hypoxia (1). Indeed, as little as 10 min of anoxia has been shown to be sufficient to induce significant biochemical alterations (2). Expression of phospho-Akt (protein kinase B) in colorectal tumors was found to be completely absent from surgically resected specimens where there had been an interruption to the blood supply of 20 min or more despite being present in the same tumor sampled earlier by biopsy (1). In breast cancer, the size of the sample has also been found to be of importance, with loss of pERK1/2 expression occurring in larger specimens (3). This was likely to be the consequence of the slow penetration of formalin and delays in the formation of stabilizing cross links between formaldehyde and proteins, which would have prevented their degradation (4). Reliance on surgically excised specimens for accurate readouts of tumor biology could, therefore, be risky (5).

If these findings were replicated in endometrial cancer, they would potentially call into question results from earlier window studies that determined drug efficacy on the basis of immunohistochemical expression of proteins in the hysterectomy specimen (6–8). Many of these lacked contemporaneous control groups for comparison and when these have been incorporated, reductions in biomarker expression in both the active drug and untreated arms suggest that reduced protein expression may be common to all surgically excised malignancies (9).

This study sought to determine whether there is a significant difference in immunohistochemical expression of commonly studied biomarkers in endometrial cancer window studies, including Ki-67, phosphorylated markers of the PI3K-Akt-mTOR and insulin signaling pathways and hormone receptors between an endometrial biopsy taken immediately prior to the start of surgery and the hysterectomy specimen. Differences in expression of endometrial cancer stem cell markers were also explored as these are likely to be increasingly used in future window studies as surrogate outcome measures. The impact of intra-tumoral hypoxia and delays in fixation of tissues on protein expression were also studied.

## Materials and Methods

### Patient and Tissue Selection

Tumor tissue was obtained from patients recruited into PREMIUM, a placebo-controlled, randomized trial of pre-surgical metformin for the treatment of atypical hyperplasia and endometrioid adenocarcinoma of the endometrium at five hospitals in the North West of England (10). The study found no effect of metformin on endometrial cancer cell proliferation, as determined by Ki-67 expression, with short-term treatment. The trial was approved by the North West Research Ethics Committee (14/NW/1236) and prospectively registered on the UK (ISRCTN 88589234) clinical trial database. All participants provided written, informed consent. Samples included an endometrial biopsy taken with a vacuum aspiration device immediately prior to the start of surgery and representative tumor blocks taken from the hysterectomy specimen itself. Patients with two matched samples taken on the same day were included in the final analysis, regardless of the allocated trial treatment arm.

After being obtained, the endometrial biopsies were immediately formalin fixed in theater and subsequently embedded in paraffin. The hysterectomy specimen was either placed straight away in formalin or was transferred dry to the local pathology department for a directed tumor biopsy before being fixed and paraffin embedded. The tumor biopsy was used for other research projects. Four-micrometer sections were cut from the tumor block using a microtome and mounted onto histological glass slides before undergoing immediate Ki-67 immunohistochemistry. This slide preparation technique was used as it has been previously demonstrated to be the most reliable and reproducible method for quantifying Ki-67 expression (11). In order to conserve tumor tissue, expression of all other markers was determined on tumor microarrays (TMAs). These were constructed using triplicate cores from representative areas of the tumor identified by an experienced gynecological histopathologist (JS) on hematoxylin and eosin stained slides.

### Immunohistochemistry

Immunohistochemistry was performed using the Leica Bond Max (Leica Biosystems, Wetzlar, Germany) and heat-induced epitope retrieval, unless otherwise stated. Full details of the antibodies and conditions used are given in Table 1. Primary antibody incubation was for 1 h with the exception of the hormone receptors where incubation was for 20 min. Primary antibody detection was performed using the Refine Detection Kit (Leica Biosystems), which utilizes a rabbit anti-mouse IgG secondary antibody and anti-rabbit poly-HRP IgG antibody and includes 3,3'-diaminobenzidine (DAB) as a chromogen. Staining for hormone receptors was performed in the clinical histopathology laboratory at Manchester University NHS Foundation Trust, using the automated Ventana BenchMark ULTRA IHC / ISH Staining Module (Ventana, Tucson, AZ, USA) and a horseradish peroxidase linked secondary antibody, with DAB as a chromogen and a substrate and copper enhancer. Counterstaining of all slides was performed using hematoxylin and negative (isotype) and positive controls were included for each antibody (Table 1).

**Table 1:** Antibodies and conditions used for immunohistochemistry.

**Antibody**	**Manufacturer**	**Catalog number**	**Dilution**	**Host**	**Antigen retrieval**	**Blocking step?**	**Positive control tissue**
Ki-67 (MIB-1 clone)	Dako	X0931	1:100	Monoclonal mouse	EDTA pH9	Included-casein	Tonsil
pAkt (Ser 473)	Cell signaling	#4060	1:50	Rabbit monoclonal	EDTA pH9	Not included	Breast cancer
p4EBP1 (Thr37/46)	Cell signaling	#2855	1:800	Rabbit monoclonal	EDTA pH9	Included	Colon cancer
pIR (Y1361)	Abcam	ab60946	1:1000	Rabbit polyclonal	EDTA pH9	Included	Placenta
pIGF1R	Abcam	ab39398	1:50	Rabbit polyclonal	Citrate buffer pH6	Included	Placenta
ER (SP1)	Ventana	790–4,324	RTU	Rabbit monoclonal	EDTA pH 8.4	Included-ultraviolet	Uterus
PR (1E2)	Ventana	790–2,223	RTU	Rabbit monoclonal	EDTA pH 8.4	Included-ultraviolet	Uterus
CD133	Miltenyi	130-090-422	1:25	Mouse monoclonal	Citrate buffer pH6	Included-casein	Colon cancer
ALDH	BD Biosciences	BD 611194	1:100	Mouse monoclonal	EDTA pH9	Included-casein	Liver
HIF-1α	BD Biosciences	BD 610959	1:50	Mouse monoclonal	EDTA pH9	Not included	Tonsil
CA-IX	Novus	NB100-417	1:2000	Rabbit polyclonal	EDTA pH9	Not included	Renal cell carcinoma

*P4EBP1, phospho-4EBP1; pIR, phospho-insulin receptor; pIGF1R, phospho-insulin like growth factor 1 receptor; ER, estrogen receptor; PR, progesterone receptor; ALDH, aldehyde dehydrogenase; RTU, ready to use; EDTA, Ethylenediaminetetraacetic acid*.

### Immunohistochemical Scoring

Slides were digitized using the Leica SCN400 Slide Scanner (Leica Microsystems, Wetzlar, Germany). Semi-automated scoring was performed using Definiens Developer software, in which an optimized solution is created to accurately identify staining of different intensity and is applied to manually selected endometrial cancer glands. The correct classification of staining within cells was checked manually by two independent researchers (SK and ZM), blinded to sample type. One researcher scored all tumors, while the second independently scored a proportion (20%) to ensure consistency. The use of the semi-automated Definiens Developer software to quantify immunohistochemical expression was more time efficient than manual scoring and associated with greater reliability and reproducibility of the scores obtained (11).

Quantification of Ki-67 staining was performed using the hot spot approach, in which the three areas of greatest Ki-67 expression across the whole slide were selected at x10 magnification, in accordance with previously published recommendations (11). The percentage of positively stained nuclei was recorded, regardless of staining intensity.

For all other markers, all malignant glands within the triplicate cores on the TMAs were scored in their entirety. With the exception of the cancer stem cell activity markers, CD133 and ALDH, staining was quantified using an H-score, which is the product of staining intensity (0 = none, 1 = weak, 2 = moderate, 3 = strong) and the percentage of cells of that intensity and has a maximum value of 300. Cells were regarded as positive if there was evidence of staining within the nucleus only (ER, PR, and HIF1α), nucleus and cytoplasm (pAkt, p4EBP1) or at the cell membrane and/or cytoplasm (pIR, pIGF1R, CA-IX). CD133 and ALDH were scored as the percentage of cells with positive apical membrane or cytoplasmic staining, respectively, regardless of staining intensity, in keeping with previous work (12, 13).

### Data Collection

Demographic and pathological data were collected by interview and from electronic and paper medical records. Where pathology services were off site, specialist gynecological pathologists were not available to perform directed tumor biopsies. Instead, the hysterectomy specimen was placed directly into formalin and potentially remained in the fixative for longer than those specimens removed at centers where pathologists were present onsite. Day of surgery was included as a surrogate marker of duration of exposure of the hysterectomy specimen to formalin, with specimens removed at the end of the working week placed in the fixative for longer due to the weekend break.

### Statistical Analysis

Data were summarized using means and standard deviations. The immunohistochemical scores of endometrial biopsies and corresponding hysterectomy specimens were compared using Wilcoxon signed-rank test. Inter-observer variability was assessed using the intra-class correlation coefficient. Comparisons of continuous, normally distributed data were performed using the Student *T*-test and of ordinal variables by Pearson's correlation coefficient. A *p* ≤ 0.05 was considered statistically significant, with asterisks used to denote significant results as ^*^p ≤ 0.05, ^**^p ≤ 0.01, ^***^p ≤ 0.001 and ^****^p ≤ 0.0001. The statistical analysis was conducted using SPSS version 23, Stata version 14 and Graph Pad Prism 7.

## Results

Matched endometrial biopsies and hysterectomy specimens of sufficient size and quality to be suitable for analysis were available for 75 patients. Assessment of inter-observer variability found excellent agreement between the two scorers with kappa values of 0.846 and 0.758 for the assessment of IHC staining in endometrial biopsies and hysterectomy specimens, respectively.

### Effect of Tumor Sampling Technique on Commonly Studied Biomarkers in Endometrial Cancer

**Figure 1:**
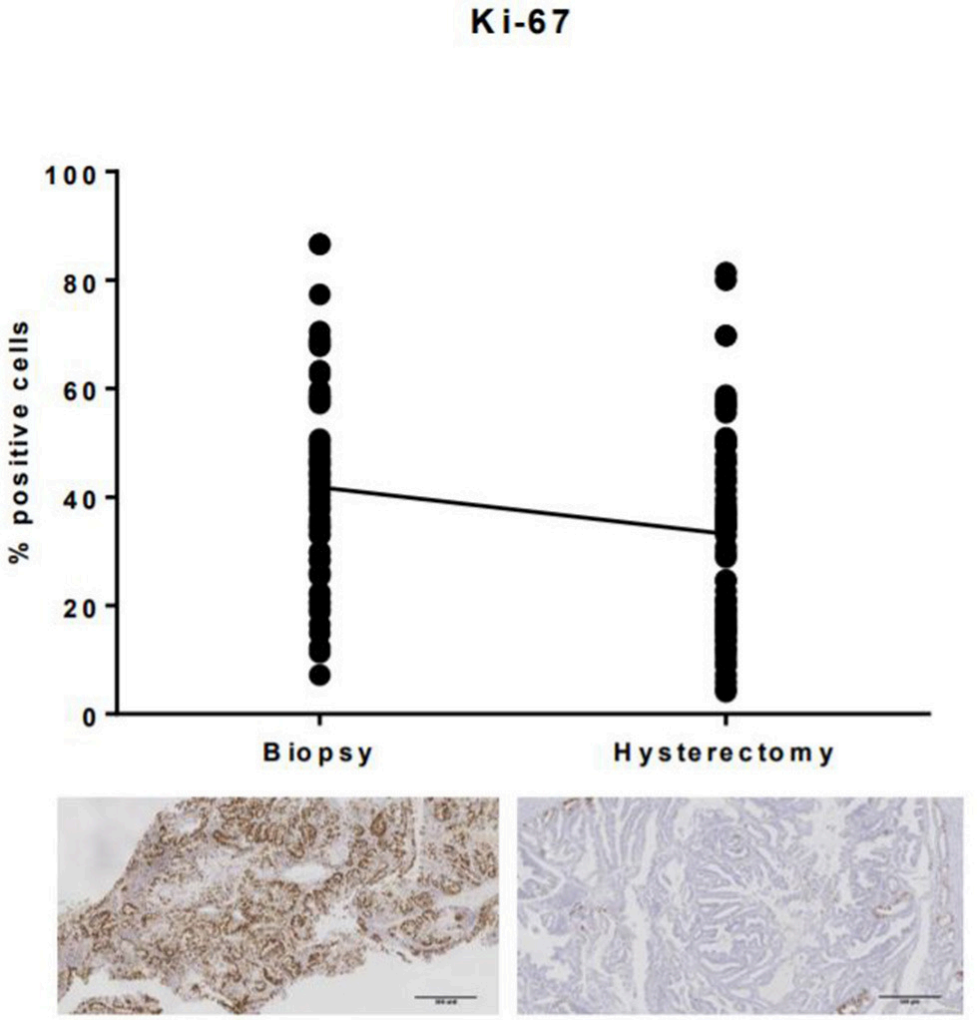
Ki-67 expression in endometrial biopsies and matched hysterectomy specimens. There was a significant difference in immunohistochemical expression of Ki-67 between endometrial biopsies and the corresponding hysterectomy specimen, with expression, on average, 8.6% (SD 15.9) lower in the surgically excised tumor sample.

Ki-67 expression was significantly lower in the hysterectomy specimen compared with the corresponding endometrial biopsy (*p* < 0.0001, Figure 1). Mean Ki-67 expression in the endometrial biopsy was 41.8% (SD 18.6%) compared with 33.2% (SD 18.7%) in the hysterectomy specimen.

**Figure 2:**
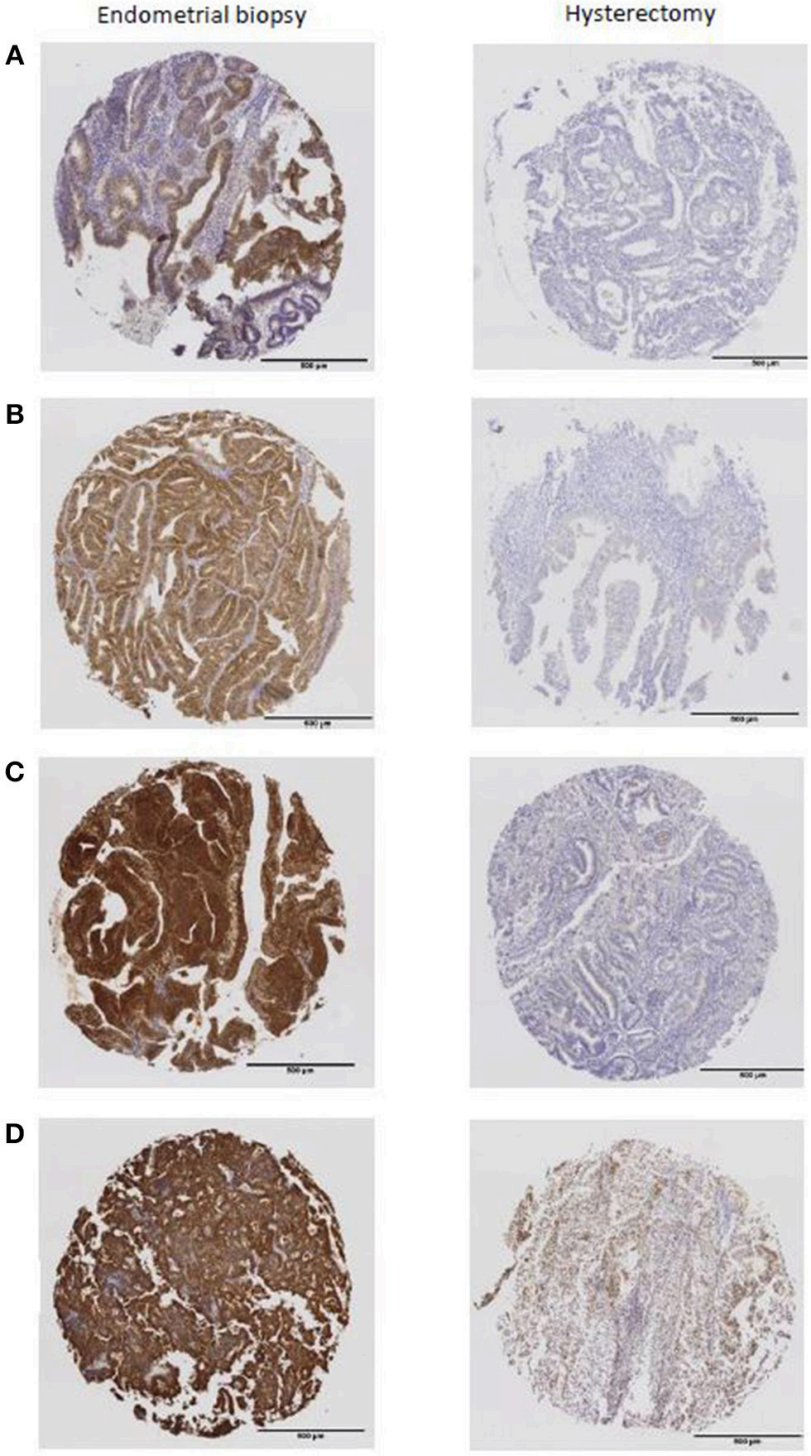
The expression of phosphorylated markers present in the endometrial biopsy were almost completely absent from the hysterectomy specimen **(A)** pAkt, **(B)** p4EBP1, **(C)** pIR, **(D)** pIGF1R.

A significant reduction in expression of phosphorylated markers of the PI3K-Akt-mTor and insulin signaling pathways was also found in the hysterectomy specimen (all *p* < 0.0001). The mean H-score for pAkt in the endometrial biopsy compared with the hysterectomy specimen was 42.6 (SD 40.0) vs. 3.3 (SD 4.8), 79.3 (SD 40.7) vs. 6.7 (SD 21.5) for p4EBP1, 200.8 (SD 38.2) vs. 55.1 (SD 50.9) for pIR and 221.8 (SD 40.1) vs. 126.2 (SD 82.7) for pIGF1R. In many cases, expression of markers was completely lost in the hysterectomy specimen (Figure 2).

**Figure 3:**
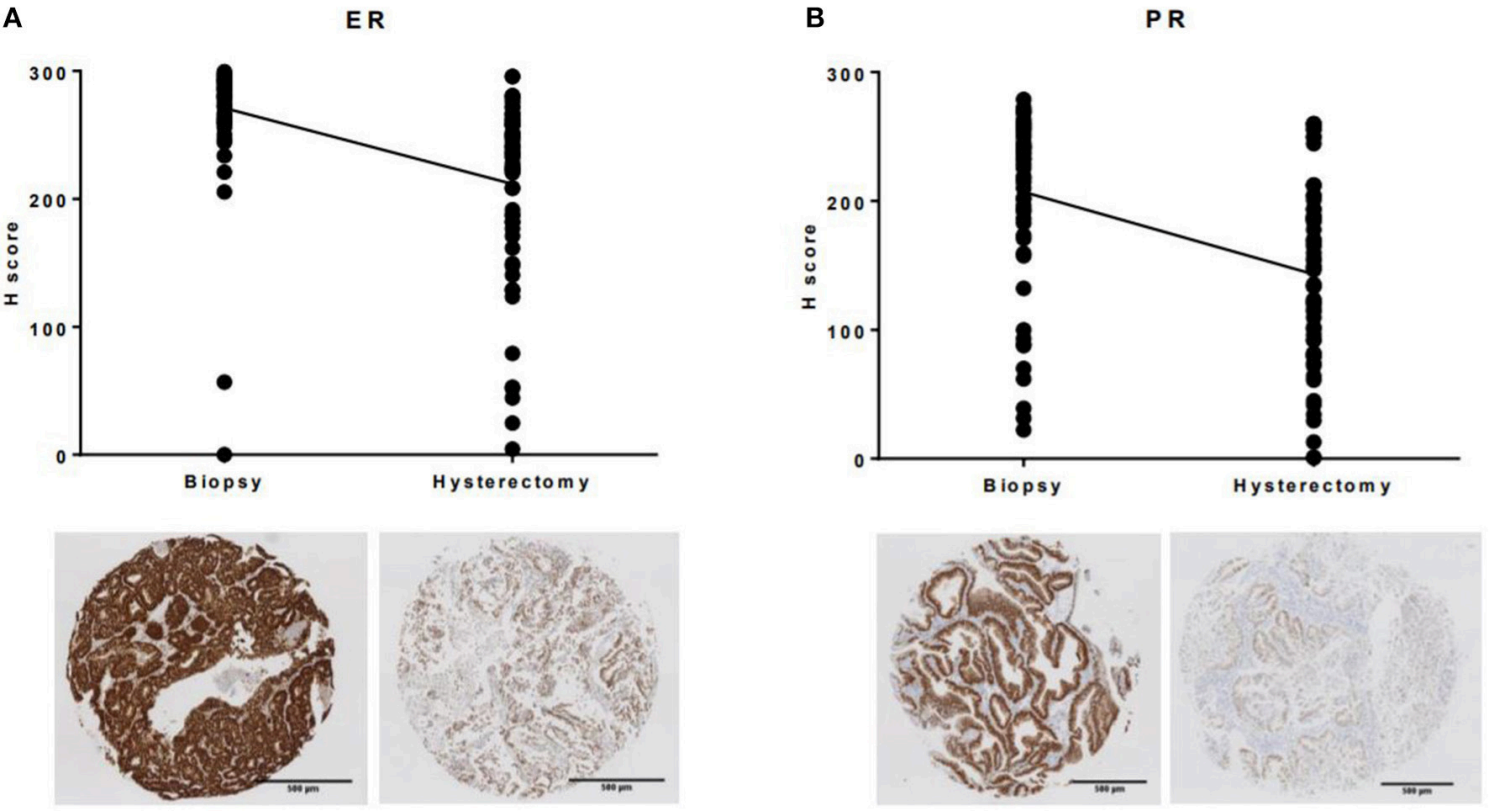
Immunohistochemical expression of ER and PR was significantly lower in the hysterectomy specimen compared to the matched endometrial biopsy **(A)** ER, **(B)** PR.

Similar results were found when expression of the hormone receptors ER and PR were compared between the two tumor sampling techniques (Figure 3). The mean H-score for ER expression decreased from 271 (SD 48.2) in the endometrial biopsy to 211.6 (SD 69.5) in the hysterectomy specimen (*p* < 0.0001), whilst PR expression decreased from 206.9 (SD 67.1) to 143.1 (SD 69.7, *p* < 0.0001). Despite lower expression of the estrogen receptor in the hysterectomy specimen, there was no discrepancy in overall ER status between the two tumor samples. This was not the case when expression of the progesterone receptor was considered; loss of PR expression in the hysterectomy specimen resulted in the misclassification of two out of 63 tumors (3.2%) as being PR negative.

In contrast to the above, expression of markers of cancer stem cell activity, CD133 and ALDH, were unaffected by sampling technique. Mean expression of CD133 was 3.3% (SD 4.7%) in the endometrial biopsy and 2.7% (SD 3.7%) in the hysterectomy specimen (*p* = 0.48). There was a non-significant increase in ALDH expression in the hysterectomy specimen, with a mean of 64.8% (SD 24.4%) of cells staining positive compared with 58.4% (SD 26.3%) in the endometrial biopsy.

The magnitude of loss of expression of Ki-67, pAkt, p4EBP1, pIR, pIGF1R, ER, and PR in the hysterectomy specimen was closely correlated with baseline expression in the endometrial biopsy. The higher the expression in the endometrial biopsy, the greater the loss of expression in the hysterectomy specimen (all *p* ≤ 0.001).

The extent to which loss of expression of each individual biomarker was associated with loss of expression of the other biomarkers was examined (Table 2). Loss of expression of p4EBP1 in hysterectomy specimens was associated with significant reductions in expression of Ki-67 (*r* = 0.24, *p* = 0.05, 68 patients) and pIGF1R (*r* = 0.32, *p* = 0.007, 71 patients). Loss of expression of pIR correlated with a reduction in expression of pAkt (*r* = 0.23, *p* = 0.05, 70 patients), whilst loss of expression of pIGF1R was associated with reductions in expression of ER (*r* = 0.29, *p* = 0.02, 63 patients) and PR (*r* = 0.36, *p* = 0.004, 61 patients). Loss of PR expression also correlated with loss of expression of pIR (*r* = 0.33, *p* = 0.008, 62 patients), pIGF1R (*r* = 0.36, *p* = 0.004, 61 patients) and, in particular, ER (*r* = 0.65, *p* < 0.0001, 61 patients).

**Table 2:** Pairwise correlation matrix of differences in biomarker expression between endometrial biopsies and hysterectomy specimens.

Ki-67 diff						
0.14 0.2665	pAkt diff					
0.24 0.05^*^ 68	0.03 0.7869	p4EBP1 diff				
−0.02 0.9067	0.23 0.05^*^ 70	0.22 0.0672	pIR diff			
0.17 0.1666	−0.07 0.5769	0.32 0.007^**^ 71	0.10 0.4272	pIGF1R diff		
0.13 0.3360	−0.04 0.7363	−0.14 0.2763	0.22 0.08 64	0.29 0.02^*^ 63	ER diff	
0.17 0.2059	0.03 0.8361	0.12 0.3662	0.33 0.008^**^ 62	0.36 0.004^**^ 61	0.65 <0.0001^****^ 61	PR diff

*Results are presented as Pearson's correlation coefficient, p-value and number of samples compared*.

* *p ≤ 0.05*,

** *p ≤ 0.01*,

**** *p ≤ 0.0001*.

Loss of biomarker expression in the hysterectomy specimen was compared against clinico-pathological variables (Table 3). Loss of expression was not associated with age, BMI, exposure to metformin or placebo, depth of myometrial invasion or need for adjuvant therapy (all *p* > 0.05).

**Table 3:** Correlation between loss of expression in hysterectomy specimen and clinico-pathological variables.

	**Ki-67 diff**	**pAkt diff**	**p4EBP1 diff**	**pIR diff**	**pIGF1R diff**	**ER diff**	**PR diff**
**AGE**							
<60 years	9.4 (16.7)	24.2 (24.2)	55.9 (45.0)	138.6 (62.9)	105.3 (102.4)	100.7 (69.3)	79.9 (86.7)
≥60 years	8.2 (15.7)	45.1 (42.2)	78.9 (46.8)	149.7 (60.2)	89.4 (99.8)	42.1 (68.1)	56.8 (88.3)
p value	0.78	0.01^*^	0.06	0.49	0.54	0.004^**^	0.34
**BMI**							
<30kg/m^2^	6.3 (16.7)	43.4 (46.7)	65.5 (53.6)	149.0 (58.4)	109.6 (96.0)	61.1 (57.1)	48.5 (81.0)
≥30kg/m^2^	10.2 (15.3)	35.9 (33.1)	76.4 (42.2)	144.6 (62.9)	83.8 (102.8)	58.4 (83.0)	74.5 (91.8)
*p*-value	0.33	0.47	0.36	0.76	0.28	0.88	0.24
**GRADE**							
1+2	9.5 (15.2)	39.6 (35.2)	72.7 (44.3)	147.3 (61.2)	92.1 (98.5)	60.8 (59.9)	68.1 (67.7)
3	3.5 (18.9)	35.0 (54.5)	67.9 (62.0)	141.4 (60.6)	106.0 (112.3)	52.7 (127.4)	59.8 (70.5)
*p*-value	0.34	0.79	0.8	0.76	0.7	0.85	0.76
**STAGE**							
1+2	9.1 (16.4)	43.1 (40.0)	73.2 (46.5)	145.8 (61.5)	95.7 (97.9)	63.2 (59.3)	71.4 (84.4)
3	4.8 (11.7)	15.6 (19.7)	63.7 (52.7)	149.3 (59.3)	87.2 (117.1)	36.7 (132.9)	36.4 (87.6)
p value	0.38	0.002^**^	0.6	0.86	0.82	0.57	0.32
**DEPTH OF MYOMETRIAL INVASION**							
<50%	8.2 (15.4)	37.7 (36.8)	72.8 (54.0)	140.5 (71.7)	76.2 (107.8)	60.6 (57.8)	65.6 (98.7)
≥50%	9.1 (18.0)	42.3 (44.1)	69.6 (37.5)	157.0 (35.8)	117.0 (83.6)	61.2 (95.5)	63.2 (72.1)
p value	0.83	0.66	0.77	0.21	0.08	0.98	0.92
**LVSI**							
Absent	9.6 (15.4)	42.5 (36.4)	79.4 (47.4)	150.0 (64.4)	91.9 (102.1)	63.1 (59.3)	70.6 (94.8)
Present	5.4 (18.7)	32.3 (45.9)	50.3 (44.2)	139.2 (50.4)	90.0 (100.2)	54.8 (105.4)	46.9 (69.7)
*p*-value	0.42	0.38	0.02^*^	0.46	0.94	0.76	0.31
**LN METS**							
Absent	8.9 (16.4)	41.2 (39.9)	73.4 (46.0)	148.2 (59.6)	94.0 (98.4)	60.5 (74.2)	65.0 (92.0)
Present	2.7 (13.4)	21.6 (31.4)	46.8 (71.4)	133.0 (75.2)	63.7 (132.0)	65.0 (82.0)	60.9 (34.2)
p value	0.43	0.2	0.46	0.65	0.61	0.91	0.85
**ADJUVANT THERAPY**							
No	8.7 (13.8)	41.4 (37.5)	70.4 (48.9)	139.2 (69.2)	82.4 (104.2)	56.9 (59.0)	59.9 (97.3)
Yes	8.3 (19.2)	37.1 (42.2)	72.9 (47.7)	156.6 (46.9)	103.4 (96.6)	65.4 (89.7)	71.0 (78.9)
p value	0.93	0.67	0.83	0.21	0.39	0.67	0.63

*Results are presented as mean difference in expression between endometrial biopsy and hysterectomy specimen (SD), LN mets lymph node metastases*.

* *p ≤ 0.05*,

** *p ≤ 0.01*.

### Impact of Poor Fixation on Loss of Immunohistochemical Expression

In order to investigate the extent to which delays in achieving adequate tissue fixation were responsible for loss of immunohistochemical expression in the hysterectomy specimen, differences in staining were correlated with specimen characteristics and tissue handling (Table 4). No clear association was found between loss of expression and tumor size, specimen weight, day of the week on which the surgery was performed or whether pathology services were located on- or offsite (all *p* > 0.05). The difference in expression between the endometrial biopsy and hysterectomy specimen was smaller if the uterus was removed by laparotomy than total laparoscopic hysterectomy, although the result was not statistically significant. Open surgery is generally associated with a shorter hypoxic time for the uterus than minimal access techniques. There was a trend toward smaller differences in immunohistochemical expression between endometrial biopsies and the hysterectomy specimen if the uterus had been bisected prior to being placed in formalin, although, with the exception of p4EBP1, this did not reach statistical significance. Whilst there was no overall correlation between loss of expression and tumor-serosal distance, this relationship was strengthened when only unopened uteri were considered.

**Table 4:** Correlation between loss of immunohistochemical expression and specimen characteristics and tumor handling.

	**Ki-67 diff**	**pAkt diff**	**p4EBP1 diff**	**pIR diff**	**pIGF1R diff**	**ER diff**	**PR diff**
Type of hysterectomy							
TLH	9.2 (19.0)	43.9 (41.8)	81.5 (49.0)	139.7 (62.3)	112.4 (101.3)	62.1 (66.7)	74.9 (98.6)
TAH	7.9 (14.6)	39.1 (38.2)	67.9 (47.9)	156.8 (53.8)	79.0 (102.4)	59.3 (81.4)	57.2 (84.2)
*p-*value	0.77	0.64	0.27	0.25	0.19	0.89	0.48
Uterus bisected							
No	10.1 (14.5)	36.2 (42.1)	84.7 (38.8)	148.7 (65.4)	114.9 (92.9)	63.8 (84.4)	71.8 (99.5)
Yes	7.1 (17.2)	41.4 (35.5)	59.5 (51.6)	144.0 (56.7)	74.5 (104.2)	54.9 (59.8)	53.7 (70.9)
*p*-value	0.44	0.58	0.02^*^	0.75	0.08	0.63	0.4
Tumour-serosal distance (mm)	−0.06	0.01	−0.01	0.09	−0.03	0.07	−0.01
	0.66	0.95	0.96	0.45	0.79	0.63	0.94
	65	65	68	68	68	59	58
Tumour-serosal distance (unopened specimens, mm)	−0.15	−0.13	−0.06	0.15	0.04	0.11	0.03
	0.43	0.47	0.76	0.41	0.81	0.56	0.86
	31	31	32	32	32	29	30
Tumour size (largest dimension, mm)	−0.01	−0.34	0.07	−0.09	0.11	−0.05	−0.11
	0.94	0.01^**^	0.62	0.52	0.43	0.73	0.48
	51	52	54	54	55	46	43
Specimen weight (g)	0.08	−0.29	−0.07	−0.01	0.06	0.02	−0.11
	0.57	0.05^*^	0.64	0.97	0.66	0.88	0.46
	52	50	52	52	51	45	44
Day of surgery	0.12	0.01	−0.04	−0.11	−0.04	−0.18	−0.21
	0.35	0.97	0.75	0.35	0.73	0.16	0.12
	66	67	70	70	70	61	60
Pathology service							
On site Off site p value	9.2 (16.3) 3.5 (11.3) 0.23	41.5 (40.0) 17.7 (16.4) 0.006^**^	69.6 (47.5) 91.1 (42.0) 0.21	146.3 (57.1) 146.5 (90.5) 1.00	90.4 (101.6) 132.5 (82.6) 0.25	53.2 (71.7) 103.7 (72.1) 0.10	56.5 (87.3) 113.5 (78.8) 0.09

*Results are presented as mean difference in expression between endometrial biopsy and hysterectomy specimen (SD) or Pearson's correlation coefficient, p-value, number of samples compared*.

* *p ≤ 0.05*,

** *p ≤ 0.01*.

### Impact of Hypoxia on Loss of Immunohistochemical Expression

**Figure 4:**
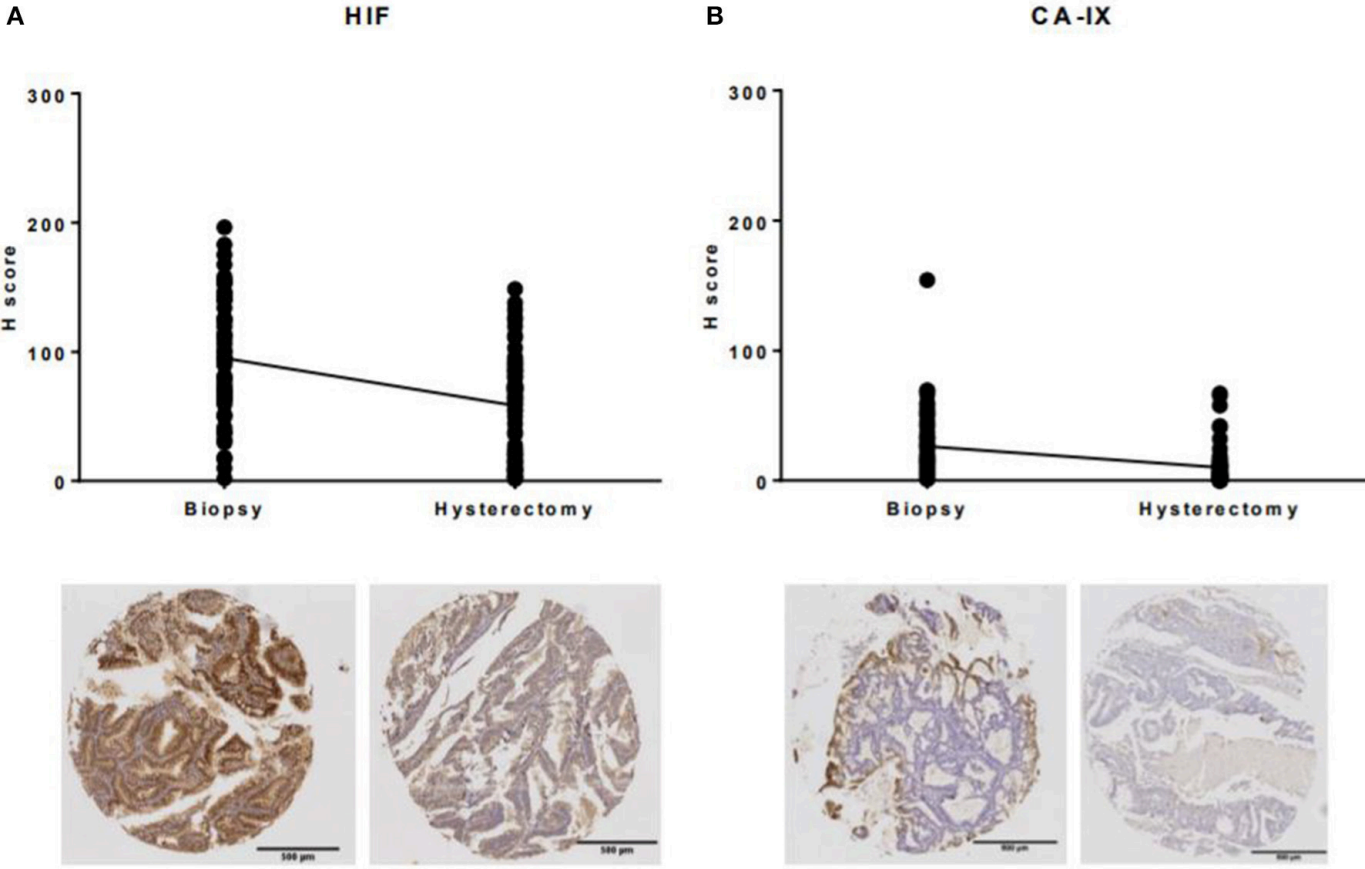
Expression of hypoxia markers in endometrial biopsies and corresponding hysterectomy specimens. **(A)** The mean H-score for HIF-1α decreased from 95.1 (SD 46.9) in the endometrial biopsy to 58.2 (SD 39.9) in the hysterectomy specimen, **(B)** the mean H-score for CA-IX decreased from 26.2 (SD 26.9) to 10.1 (SD 15.5).

To determine the impact of hypoxia of the uterine tissues on loss of immunohistochemical expression of commonly studied biomarkers in endometrial cancer window studies, expression of HIF-1α and its downstream effector CA-IX were compared in the hysterectomy specimen to the corresponding endometrial biopsy. Paradoxically, despite the uterine blood supply being clamped for at least 20 min during surgical excision and obvious discoloration of the uterus as a result, expression of both hypoxia markers was significantly lower in the hysterectomy specimen than in the endometrial biopsy (both *p* < 0.0001, Figure 4).

## Discussion

The immunohistochemical expression of commonly studied biomarkers in endometrial cancer window studies, including Ki-67, phosphorylated markers of the PI3K-Akt-mTOR and insulin signaling pathways and hormone receptors, is significantly lower in the hysterectomy specimen compared with an endometrial biopsy taken immediately prior to the commencement of surgery. In contrast, expression of markers of cancer stem cell activity, such as CD133 and ALDH, are unaffected by tumor sampling technique. Investigation of the extent to which loss of protein expression was due to surgically induced hypoxia was hampered by lower levels of immunohistochemical staining of hypoxia inducible factor and its downstream effector in the hysterectomy specimen. This was despite observational evidence of reduced perfusion for over 30 min, which would normally result in a significant increase in expression of these hypoxia inducible proteins (14, 15). The extent to which biomarker expression was lost in the hysterectomy specimen was closely related to baseline expression of these proteins in the endometrial tumor and was reduced if the uterus was bisected before being placed in formalin.

These findings suggest that delays in achieving adequate fixation of tissues are of critical importance in driving loss of detectable protein expression in surgically excised specimens. Formalin penetrates tissues at a rate of only 1 mm/h, meaning that it can be several hours before it has reached and fixed tumor tissue located within the uterine cavity (2). In breast cancer, a time interval of 16 h or more between surgical excision of a tumor and its fixation has been shown to be sufficient to result in a significant reduction in expression of Ki-67 (16). Similarly, prolonged fixation (>2 days) has also been shown to be detrimental to the expression of proliferation markers, highlighting the importance of developing and adhering to guidelines of the optimization of pre- and intra-fixation parameters. As the results of this study have also shown, immediate dissection and sectioning of large surgical specimens is necessary to ensure proper fixation (17).

Whilst the insensitivity of CD133 and ALDH to tumor sampling technique may initially appear surprising, hypoxia has previously been shown to enrich for cancer stem cells (18). Expression of both of these markers at both the mRNA and protein level as well as the proportion of CD133^+ve^ and ALDH^high^ cells detected by flow cytometry is increased in response to the growth of glioblastoma and ovarian cancer cell lines in 1% oxygen (18–20). Any loss of expression through poor fixation is, therefore, likely to be counteracted by the hypoxia-induced increase in expression of these markers within endometrial cancer cells. The fact that expression of the hypoxia markers HIF-1α and CA-IX were significantly lower in the hysterectomy specimens compared with the endometrial biopsies suggests that the cancer stem cell markers studied were more stable at the protein level and resistant to delays in fixation.

In order to avoid the issue of loss of protein expression in hysterectomy specimens, the use of endometrial biopsies for post-treatment immunohistochemical analysis is to be encouraged, as occurred in the aforementioned PREMIUM trial (10). The smaller size of a biopsy sample means that fixation can occur much more rapidly and, as a consequence, more accurately reflects tumor biology. The findings reported here mirror those seen in breast cancer, where immunohistochemical expression of pAkt and pERK1/2 was found to be significantly lower in surgically excised specimens than in core-cut biopsies and, indeed, was found to be almost absent in a number of cases (3). Whilst Pinhel et al. found no significant difference in expression of hormone receptors in breast cancers removed surgically or sampled by biopsy, their analysis was based on only 29 samples and there was a trend toward lower expression of ER in excised specimens. From a clinical point of view, the concern has been raised that inappropriate decisions regarding adjuvant treatment may be made if hormone receptor status is determined solely in surgical specimens. It has been estimated that up to 9% of breast cancer cases may be denied the benefits of hormone therapy due to false negative immunohistochemistry results (5). For window studies, smaller changes in protein expression are used to determine drug efficacy, meaning that the hysterectomy specimen can no longer be considered adequate to be used in the assessment of primary and secondary outcomes in these kinds of trials in endometrial cancer. This potentially calls into question the results of earlier studies which reported a reduction in immunohistochemical staining of Ki-67 and phosphorylated markers of the PI3K-Akt-mTOR and MAPK/ERK pathways with exposure to metformin and anastrazole in the pre-surgical window (6, 7, 21). Future studies should be designed to compare expression of biomarkers in endometrial biopsies taken prior to and following drug treatment, with the latter performed immediately prior to the start of the hysterectomy procedure as in the case of the PREMIUM study (10).

Whilst concerns may be raised that “blind” endometrial biopsies risk sampling error by failing to analyze a representative proportion of the tumor, this risk may be minimized by obtaining a generous sample under general anesthetic. In the current study, only cases for whom a representative endometrial biopsy was taken immediately prior to the start of the hysterectomy procedure were included. For 75 of the 88 women (85%) participating in the PREMIUM trial, the endometrial biopsy was of sufficient size and contained tumor of similar morphology to that of the hysterectomy specimen, as determined by a specialist gynecological pathologist. In three cases it was not possible to obtain an endometrial biopsy due to cervical stenosis and in the remaining 10 cases, the biopsy was either too small or not representative of the tumor overall. It would therefore appear to be both feasible and appropriate to use endometrial biopsies rather than the hysterectomy specimen for analysis of trial outcomes in window studies. A disadvantage is that it does not allow assessment of the myometrium, and may contain minimal stroma or normal endometrium for comparison, although this may not be relevant if the effect of drugs on the tumor glandular component only is being assessed.

The strengths of this study include the comparison of a range of biomarkers used as outcome measures in endometrial cancer window studies in a large sample of endometrial biopsies and matched hysterectomy specimens. Scoring was performed using semi-automated software, which has been shown to be more reliable and reproducible than manual scoring (11).

Only limited data were available regarding the handling of tissues once they had been surgically removed from the body; in particular, details of the time interval before the specimen was placed in formalin (the cold ischemia time) and the length of time it remained in the fixative before sectioning were not recorded. Surrogate measures were therefore used in their place to investigate the relationship between loss of immunohistochemical staining and delays in or prolongation of fixation, including specimen and tumor size and tumor-serosal distance. The extent to which these variables correlate with tumor fixation have not, however, been determined. Whilst records were kept of the number of surgical specimens opened to obtain a directed tumor biopsy prior to being placed in formalin were kept, the number of uteri that were bisected but then did not have a biopsy taken due to insufficient surplus tumor were not documented. This was known to have happened on at least several occasions. The association between formalin penetration and loss of immunohistochemical staining may have been strengthened if these data had been available. It is possible to limit the cold ischemia time, particularly if fresh, frozen tissue is not required, by placing specimens directly into formalin and bisecting large specimens, such as the uterus, to reduce the time required for the fixative to penetrate deep tissues. If fresh tissue is required, ideally a pathologist should be available within the operating room to perform the necessary sampling, otherwise appropriately trained deputies should be present to prevent “research sampling” impacting clinical diagnostic assessment.

Correlation between the degree of loss of protein expression in the hysterectomy specimen and surgically induced hypoxia was not possible due to the presence of lesser degrees of staining of hypoxia markers in these samples. This may well be the result of poor fixation of these tissues. Alternative methods of determining intra-tumoral hypoxia, however, are either invasive or expensive, thereby negating their use (22). Regardless of the reason for the loss of protein expression in the hysterectomy specimen, the end result is a sample that no longer represents the tumor from which it was obtained, especially for phosphorylated markers.

## Conclusion

Immunohistochemical staining of commonly studied biomarkers in endometrial cancer window studies, including Ki-67, phosphorylated markers of key carcinogenic pathways and hormone receptors, is significantly lower in the hysterectomy specimen than in an endometrial biopsy taken immediately prior to surgery. Surgically induced hypoxia and, in particular, poor fixation of large specimens are likely to be responsible. In order for methodologically robust results to be generated from future endometrial cancer window studies, endometrial biopsies should be used for post-treatment analyses.

## Ethics Statement

This study was carried out in accordance with the recommendations of North West Research Ethics Committee (14/NW/1236) with written informed consent from all subjects. All subjects gave written informed consent in accordance with the Declaration of Helsinki. The protocol was approved by the North West Research Ethics Committee.

## Author Contributions

EC obtained funding for the study and was its Chief Investigator, designed the study, supervised study execution, contributed to data interpretation, edited the manuscript, and study guarantor. SK designed the study, recruited to the study and performed clinical and laboratory study procedures, data interpretation and wrote the manuscript. SK, ZM, and VS undertook immunohistochemical scoring on tumors annotated by JS. All authors provided critical comment and approved the final version of the manuscript.

### Conflict of Interest Statement

The authors declare that the research was conducted in the absence of any commercial or financial relationships that could be construed as a potential conflict of interest.
